# Timeliness of Colorectal Cancer Care in Veterans: The Role of Travel Distance

**DOI:** 10.1002/cnr2.70663

**Published:** 2026-08-31

**Authors:** Emma M. Bradley, Megan Shroder, Gretchen Edwards, Shannon McChesney, Colleen Kiernan, Robert S. Dittus, Christianne L. Roumie

**Affiliations:** ^1^ Department of General Surgery Vanderbilt University Medical Center & Tennessee Valley Healthcare System—Medical Center North Nashville Tennessee USA; ^2^ Geriatric Research Education and Clinical Center, and the VETWISE‐ LHS Center of Innovation Tennessee Valley Healthcare System—Veterans Health Administration Nashville Tennessee USA; ^3^ Division of Colon & Rectal Surgery, Department of Surgery Vanderbilt University Medical Center—Medical Center North Nashville Tennessee USA; ^4^ Division of Surgical Oncology, Department of General Surgery Vanderbilt University Medical Center—Medical Center North Nashville Tennessee USA; ^5^ Department of Medicine, Division of General Internal Medicine and Public Health Vanderbilt University Medical Center Nashville Tennessee USA

**Keywords:** access to care, colorectal cancer, timeliness of care, travel distance, Veteran's health

## Abstract

**Background:**

Rural patients experience healthcare disparities due to multiple factors including travel distance. However, the impact on colorectal cancer (CRC) care for Veterans remains undefined.

**Aims:**

We aimed to describe the association between distance and timeliness of care for Veterans with CRC.

**Methods and Results:**

We assembled a retrospective cohort of Veterans with Stage I–III CRC who underwent resection at a Veterans Affairs Medical Center (VAMC) between 2001 and 2018. Time from diagnosis to initiation of treatment, length of stay, 30‐day readmissions and mortality were compared based on distance (≤ 40 mi; 41–100 mi, and > 100 mi). Among 375 patients, the median travel distance was 67.9 mi and median time to treatment initiation was 33 days. In a multivariable linear regression adjusted for age, stage, year of surgery, race and co‐morbidities, the time to initial treatment was 11.8 days longer for patients > 100 mi away compared to those who live within 40 mi (CI 1.4–22.1, *p* = 0.026). No significant difference was observed for patients within 40 mi vs. 41–100 mi away (0.13 days, CI −9.4 to 9.8, 0.98). Common contributors to delays > 60 days included transitions from non‐VAMC care, cardiac optimization and additional testing or treatment. There were no associations between distance and surgical length of stay, 30‐day readmissions, or mortality.

**Conclusion:**

Travel distance > 100 mi was associated with timeliness of care for Veterans, though no differences in clinical outcomes were observed. Distance‐related outcome disparities may be mitigated through integrated, high‐volume VA care system.

## 1 Introduction

Colorectal cancer (CRC) is the second leading cause of cancer‐related death in the United States (US) [1]. For Veterans, CRC is the third most commonly diagnosed cancer comprizing over 10% of cancers within the Veterans Health Administration (VHA) [2, 3]. For cancer patients, delays in oncologic care can result in advanced disease and treatment challenges, as well as increased mortality [4, 5, 6, 7, 8].

As the largest provider of integrated cancer care in the US, the VHA represents a unique healthcare system, operating as a national single‐payer model with coordinated, longitudinal care. Timeliness and access are priorities for Veteran's healthcare. While some Veterans receive part or all their cancer care in the community, many prefer to remain in the VHA system [9, 10]. Recent studies have demonstrated that quality, safety and health outcomes are comparable with community care for diagnosis and treatment of many disease processes [11, 12, 13].

Care delay between diagnosis and the start of the treatment pathway for CRC is associated with patient specific factors such as age, race and medical comorbidities, as well as institutional center volume and region of the country [14, 15, 16]. Travel burden can contribute to delays in medical care in general, especially among rural patients [17, 18, 19]. Over the past 10 years, travel distance for rural patients to obtain both low and high‐risk surgical care has increased [20]. As complex cancer care has become increasingly centralized to higher acuity cancer centers in the US, understanding the impact of longer travel distances is valuable for all patients.

However, the relationship between travel distance and CRC treatment for Veterans is not well described. Data delineating these relationships in the VHA context is important not only for health system planning and care delivery, but also for counseling patients on their treatment options and trajectory of care. Our aim was to test the hypothesis that longer travel distance to the Veterans Administration Medical Center (VAMC) is associated with delays in care for Veterans with newly diagnosed Stage I–III CRC.

## 2 Methods

### 2.1 Study Design, Setting, and Data Sources

We assembled a retrospective cohort of Veterans who underwent colorectal resection between 2001 and 2018. The passage of the Mission Act in 2018 had a large impact on community care referrals, particularly for Veterans furthest from VAMCs [21, 22, 23]. In order to focus primarily on care administered by the VA, as well as address the possibility of a selection bias that may occur with fewer patients from longer travel distances, we did not include Veterans treated after 2018.

This cohort was derived from patients included in a local surgical quality database, which includes all Veterans undergoing colorectal surgeries at a single VAMC, an academically affiliated medical system that provides endoscopic, oncologic and major surgical care. This study was approved by the institutional review board (IRB) with a waiver of informed consent, and was conducted in accordance with the US Federal Policy for the Protection of Human Subjects and the Declaration of Helsinki.

### 2.2 Study Population

Veterans were included and reviewed using a standardized chart abstraction tool if they were above the age of 18 and underwent oncologic surgical resection for Stage I–III colorectal cancer. Veterans with Stage 0 disease and those with preoperative or intraoperative evidence of metastatic disease were excluded. We further excluded patients who underwent resection for reasons other than colon or rectal adenocarcinoma, palliative surgical resection, diversion, operation for an emergent or urgent indication (bleeding, obstruction, or perforation) or surgery within 3 days of diagnosis (indicating an urgent or emergent case).

### 2.3 Data Collection

Data was extracted using a structured chart abstraction form from the VA Computerized Patient Record System (CPRS) and Joint Legacy Viewer. Patient demographic data included Veteran age, sex, race, body mass index (BMI), and smoking status (never, unknown, prior, or current) as documented in the electronic health record. Comorbidity data included diagnosis of diabetes mellitus, chronic kidney disease (CKD, defined as an estimated glomerular filtration rate of < 60 mL/min/1.73 m^2^ without evidence of an acute kidney injury), chronic obstructive pulmonary disease (COPD, defined as most recent pulmonary function tests with FEV1/FVC < 80%), congestive heart failure (CHF, as a documented co‐morbidity or ejection fraction on echocardiogram of < 50%), and cardiovascular disease (CVD, described as prior myocardial infarction [MI], obstructive coronary artery disease with prior stent placement, peripheral arterial disease, carotid disease, or prior historical stroke or transient ischemic attack). Additional oncologic data abstracted included colonoscopy purpose (screening or symptomatic), treatment received and duration, cancer type (colon or rectal cancer) and American Joint Committee on Cancer stage. To most accurately assess burden of disease that would influence clinical care, pathologic stage was utilized for colon cancer patients and pretreatment clinical stage was used for rectal cancer patients (given the potential for a lower stage at surgery if a patient received neoadjuvant therapy).

The primary predictor was travel distance from Veteran's residence zip‐code to the VAMC, categorized as ≤ 40 mi; 41–100 mi, and > 100 mi. The cutoff of 40 mi represents the criterion for community care referral under the Veterans Choice Program [24]. Travel distance was directly related to and used as a practical proxy for rurality. To further describe rural composition, we used the US Department of Agriculture Rural–Urban Commuting Area (RUCA) codes, categorizing patients living in metropolitan or micropolitan areas as “urban” and those in small town or rural areas as “rural” [25, 26]. Distances and RUCA codes were derived using zip codes and Google mapping software. Sensitivity analyses explored alternative distance thresholds (20, 60, 80, and 120 mi) as well as urban vs. rural categorizations. These alternative groupings were also evaluated within adjusted multivariable models to assess the robustness of findings.

### 2.4 Outcomes: Time to First Definitive Treatment

The primary outcome was the number of days from CRC diagnosis, defined by the date of the pathology report for the colonoscopy or sigmoidoscopy, to treatment initiation. Treatment initiation was defined as the date of first definitive treatment (including surgery, chemotherapy, or radiation) based on tumor type, stage, and multidisciplinary tumor board recommendations. Secondary outcomes included length of stay after the index surgical operation, readmission (yes/no) within 30 days of index surgical hospitalization, and overall survival from surgery.

### 2.5 Exploratory Analysis of Factors Contributing to Care Delays

We conducted an exploratory analysis among the subgroup of patients who had more than a 60‐day interval between cancer diagnosis and treatment initiation to evaluate the factors that contributed to treatment delays. A priori we hypothesized these factors would be organized into the following groupings: (1) medical factors (including cardiac and noncardiac risk stratification or optimization, testing and treatment of other incidental findings, and other medical problems), (2) system factors (including delay in testing for oncologic staging procedures such as endoscopic ultrasound [EUS] or additional colonoscopy, scheduling issues, and care transition from outside medical facility), and (3) patient specific factors (preference, difficulty contacting, or keeping appointments).

### 2.6 Statistical Analysis

We used Chi‐squared, Kruskal–Wallis, or Mann–Whitney *U* tests in univariate comparison of proportions or medians across travel distance groups. Covariates included in the regression models were selected a priori based on clinical relevance with considerations to avoid overfitting. Using multivariable linear regression, we evaluated the relationship between travel distance and time to initiation of treatment, adjusted for age, race, year of surgery, clinical cancer stage and type, medical co‐morbidities, and colonoscopy indication. Year of surgery was modeled using a restricted cubic spline to account for nonlinear temporal trends. Results are reported as regression coefficients (*β*), which represent the number of days in time to initiation of therapy between the reference group (< 40 mi) and the other two travel distance groups (41–100 mi and more than 100 mi). *p* ≤ 0.05 was considered statistically significant, and 95% confidence intervals (CI) are reported.

For the secondary outcomes of length of stay and readmission within 30 days we used multivariable linear or logistic regressions accounting for the above covariates, respectively. To evaluate the association between categorical travel distance and the secondary outcome of all‐cause mortality, we constructed Kaplan–Meier curves and log‐rank tests. Survival data was truncated at 24 months due to the proportional hazards assumption no longer being met because of sparse data. Cox proportional hazards model, adjusted for the above covariates, evaluated the association between travel distance and survival up to 2 years.

Statistical analysis was performed with STATA version 18.0 (StataCorp, College Station, TX) and R Studio version 2023.12.1 + 402 (R Foundation for Statistical Computing, Vienna, Austria) software [27].

## 3 Results

### 3.1 Flow Chart and Patient Characteristics

**FIGURE 1:**
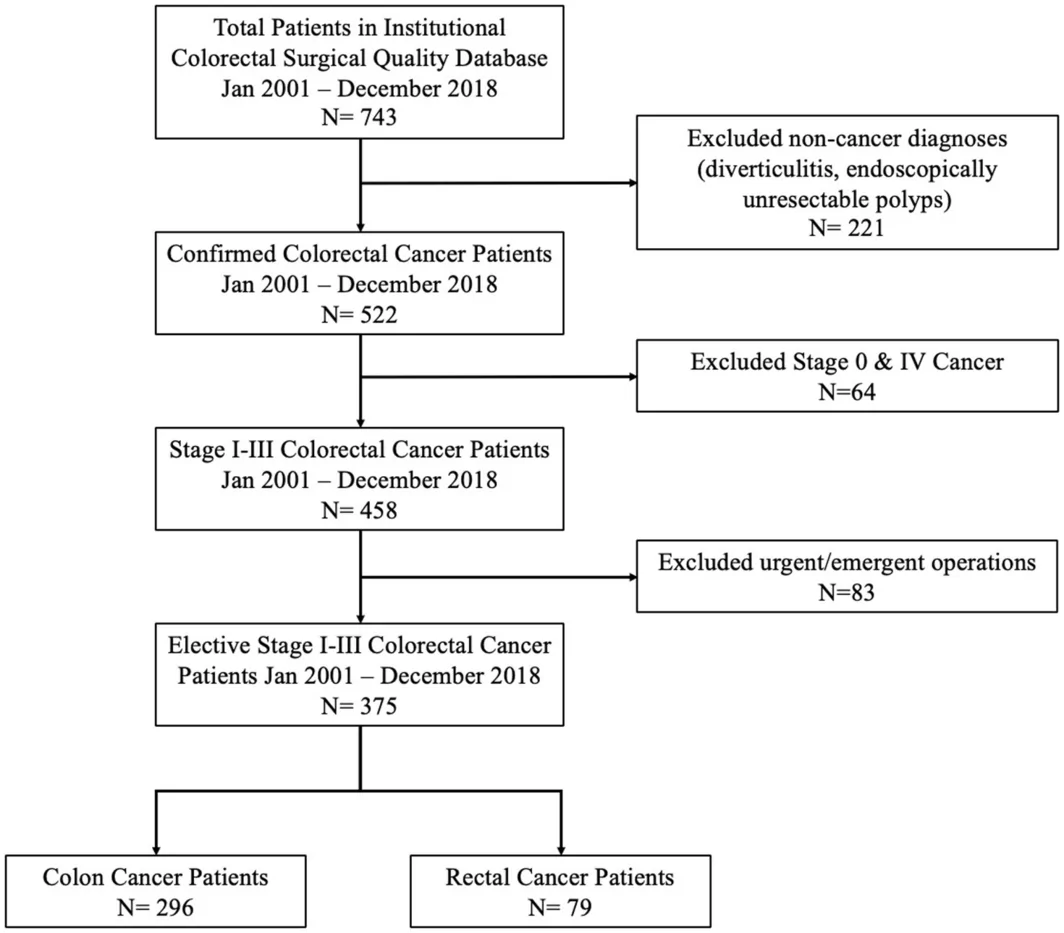
Flow diagram of Veterans included in time to initiation of treatment analysis.

Out of 743 Veterans who underwent a colorectal surgical procedure, 458 Veterans were included for surgery for Stage I–III CRC. Of these, 83 were excluded for emergency or urgent operations. There were 375 patients included in the final analytic cohort, 296 with colon and 79 with rectal cancer (Figure 1).

The median age was 66 years (interquartile range [IQR] 60–73), with most of the Veterans being male (97.1%) and White (80.3%). Most Veterans lived in urban (84.3%) versus rural (15.7%) areas. A significant proportion of Veterans (90.1%) had an ASA (American Society of Anesthesiologists Physical Status) classification of 3 or 4, indicating the presence of notable comorbidities. Among the cohort, 37.9% carried a prior diagnosis of CHF or cardiovascular disease, 27.7% with COPD, 10.1% with CKD and 35.2% with diabetes. There was a high prevalence of prior and current smokers (55.5% and 22.9%, respectively) at the time of diagnosis. Sixty percent of cancers were detected because their colonoscopy was performed for symptoms while 39.7% of colonoscopies were performed for screening purposes (Table 1).

**TABLE 1:** Study population characteristics.

Characteristic	Travel distance to Veterans Affairs medical center				*p*
	≤ 40 mi, *N* = 110 (29.3%)	41–100 mi, *N* = 155 (41.3%)	*>* 100 mi, *N* = 110 (29.3%)	Total^a^ *N* = 375	
**Patient characteristics**					
Age in years, median (IQR), years	66 (60, 73)	66 (60, 72)	65 (61, 73)	66.0 (60, 73)	0.840
Sex, *n* (%)					0.961
Male	107 (97.3)	150 (96.8)	107 (97.3)	364 (97.1)	
Female	3 (2.7)	5 (3.2)	3 (2.7)	11 (2.9)	
Race, *n* (%)					0.004
Black	27 (24.5)	19 (12.3)	7 (6.4)	53 (14.1)	
Hispanic or Latino	1 (0.9)	0 (0.0)	0 (0.0)	1 (0.3)	
Other^b^	4 (3.6)	10 (6.5)	6 (5.5)	20 (5.3)	
White	77 (70.6)	126 (81.3)	97 (88.2)	301 (80.3)	
Body mass index, median (IQR)	26.8 (7.2)	28.3 (6.7)	26.8 (6.6)	27.5 (6.7)	0.095
Rurality					> 0.001
Urban (metro/micropolitan)	110 (100)	124 (80)	82 (74.5)	316 (84.3)	
Rural (small town/rural)	0 (0)	31 (20)	28 (25.5)	59 (15.7)	
**Comorbidities**					
CKD III or higher, *n* (%)^c^	9 (8.2)	15 (9.7)	14 (12.7)	38 (10.1)	0.520
CHF and/or CVD, *n* (%)^d^	46 (41.8)	52 (33.5)	44 (40.0)	142 (37.9)	0.338
COPD, *n* (%)^e^	25 (22.7)	46 (29.7)	33 (30.0)	104 (27.7)	0.377
Diabetes, *n* (%)	44 (40.0)	46 (29.7)	42 (38.2)	132 (35.2)	0.164
Smoking status, *n* (%)					0.351
Never	24 (21.8)	28 (18.1)	29 (26.4)	81 (21.6)	
Prior	58 (52.7)	95 (61.3)	55 (50.0)	208 (55.5)	
Current	28 (25.5)	32 (20.6)	26 (23.6)	86 (22.9)	
ASA class, *n* (%)^f^					0.271
ASA 2	10 (9.3)	21 (13.6)	6 (5.5)	37 (10.0)	
ASA 3	85 (78.7)	116 (75.3)	92 (84.4)	292 (79.0)	
ASA 4	13 (12.0)	17 (11.0)	11 (10.1)	41 (11.1)	
**Cancer characteristics**					
Indication for colonoscopy, *n* (%)					0.112
Screening	37 (33.6)	71 (45.8)	41 (37.3)	149 (39.7)	
Symptoms	73 (66.4)	84 (54.2)	69 (62.7)	226 (60.3)	
Stage, *n* (%)^g^					0.654
Stage I	30 (27.3)	51 (32.9)	37 (33.6)	118 (31.5)	
Stage II	46 (41.8)	57 (36.8)	46 (41.8)	149 (39.7)	
Stage III	34 (30.9)	47 (30.3)	27 (24.5)	107 (28.8)	
Cancer type, *n* (%)					0.782
Colon cancer	86 (78.2)	125 (80.6)	85 (77.3)	296 (78.9)	
Rectal cancer	24 (21.8)	30 (19.4)	25 (22.7)	79 (21.1)	
Initial oncologic treatment					
Surgery	95 (86.4)	139 (89.7)	95 (86.4)	329 (87.7)	0.629
Neoadjuvant therapy	15 (13.6)	16 (10.3)	15 (13.6)	46 (12.3)	

^a^ Among the total cohort, median travel distance to VAMC was 67.9 mi (IQR 36.1–114).

^b^ “Other” includes Asian, American Indian or Alaska Native, Native Hawaiian or Other Pacific Islander.

^c^ CKD (chronic kidney disease Stage III or above) defined as glomerular filtration rate < 60, not representing acute kidney injury.

^d^ CHF (congestive heart failure) if documented history and/or ejection fraction < 50%. CVD (cardiovascular disease) is defined as prior myocardial infarction, obstructive coronary artery disease with stent, peripheral arterial disease, carotid disease, or prior cerebrovascular accident or transient ischemic attack.

^e^ COPD (chronic obstructive pulmonary disease) if documented history of pulmonary function tests with FEV1/FVC < 80.

^f^ American Society of Anesthesiologists (ASA) Physical Status classification.

^g^ Stage represents pathologic stage for colon cancer patients and clinical stage among rectal cancer patients.

### 3.2 Travel Distance to VAMC

Median travel distance to VAMC was 67.9 mi (IQR 36.1–114). Distance did not differ among the Veterans with colon versus rectal cancer (66.3 mi vs. 69.1 mi, *p* = 0.81). We categorized Veterans by their travel distance to the VAMC into three groups: 110 (29.3%) patients within 40 mi, 155 (41.3%) patients between 41 and 100 mi, and 110 (29.3%) patients with distance greater than 100 mi to the VAMC. Further travel distances were associated with a higher proportion of rural Veterans, comprising 0%, 20%, and 25.5% of the ≤ 40, 41–100, and > 100 mi groups, respectively (Table 1).

Comparisons between the three groups demonstrated that Veterans were more likely White if they lived > 100 mi vs. those 41–100 mi and < 40 mi (88.2 vs. 81.3 vs. 70.6%, *p* = 0.004). Otherwise, the groups were similar in characteristics (Table 1). After 2014 there were fewer patients with travel distance > 40 mi from the VAMC (65.88% vs. 72.06% prior to 2014), although this difference was not statistically significant. There was no difference in average number of medical comorbidities per Veteran before or after 2014 (1.49 vs. 1.56, *p* = 0.75) (Table S2).

### 3.3 Time Between CRC Diagnosis and Treatment Initiation

The median number of days until treatment initiation was 33 days (IQR 19–54), 30 days (IQR 19–50) for Veterans with colon cancer and 46 days (IQR 26–61) for rectal cancer. In multivariable linear regression which adjusted for age, race, year of surgery, cancer stage and type, medical co‐morbidities and indication for colonoscopy, the time to treatment was 11.77 days longer for patients with travel distance of > 100 mi compared to those who lived ≤ 40 mi (95% CI 1.38–22.15). There was no significant difference in time to treatment amongst the patients living 41–100 mi away compared to those within 40 mi (0.13 days, 95% CI −9.47–9.72). Other covariates associated with a statistically significant increase in treatment included: rectal versus colon cancer (16.87 days longer, CI 6.97–26.76), year of surgery (10.19 days longer in 2008 vs. 2005, CI 5.36–15.01 and 17.14 days longer in 2013 than 2005, CI 10.51–23.76), and colonoscopy performed for screening vs. symptoms (9.21 day difference, CI 0.72–17.70) (Table 2).

**TABLE 2:** Multivariable regression for association of distance with time to initiation of treatment for colorectal cancer.

Characteristic	*β* coefficient (days difference)	95% Confidence interval	*p*
Distance from VAMC			
40–100 vs. ≤ 40 mi	0.13	−9.47 to 9.72	0.98
≤ 40 mi	**11.77**	**1.38–22.15**	**0.03**
Age	0.40	−0.07 to 0.87	0.10
Race			
Non‐White vs. White	8.95	−2.22 to 20.11	0.12
BMI			
**≥ 30** vs. > 30	6.11	−2.77 to 14.99	0.18
Smoking status			
Current vs. never/prior	8.03	−2.63 to 18.69	0.14
CKD	−4.90	−18.72 to 8.92	0.49
CHF and/or CVD	3.55	−4.99 to 12.08	0.41
COPD	9.29	−0.02 to 18.60	0.05
Year of surgery^a^			
2008 vs. 2005	**10.19**	**5.36–15.01**	**< 0.001**
2013 vs. 2005	**17.14**	**10.51–23.76**	**< 0.001**
Cancer type			
Rectal vs. Colon	**16.87**	**6.97–26.76**	**0.001**
Cancer stage			
Stage II vs. Stage I	0.67	−9.02 to 10.36	0.89
Stage III vs. Stage I	0.70	−9.69 to 11.09	0.89
Indication for colonoscopy			
Screening vs. Symptom	**9.21**	**0.72–17.70**	**0.03**

*Note:* Bold results represent statistical significance. *R* ^2^ = 0.17; overall model *p* < 0.0001.

^a^ Year of surgery included in the model using a restricted cubic spline for nonlinearity. Reported values are for years 2008 and 2013 compared with the year 2005.

Several sensitivity analyses were also performed to assess the robustness of these findings using alternative definitions of travel distance and rurality, including thresholds of 20, 60, 80, and 120 mi, adjusted for the same covariates. These yielded results consistent with those of the primary analysis. There remained a statistically significant longer interval between diagnosis and initiation of treatment > 120 mi compared to Veterans within 40 mi, on the order of 11.49 days (95% CI 0.31–22.67). Time to initiation of treatment was approximately 8.14 days longer for patients > 80 mi away versus within 40 mi (95% CI −1.43 to 17.71), and 8.82 days longer for patients living in rural versus urban areas (95% CI −2.31 to 19.95), although these differences were not statistically significant. All five sensitivity analyses have been included in Table S1.

### 3.4 Clinical Outcomes

Select clinical outcomes are described in Table S3. Length of hospitalization did not differ by distance, with a median length of stay of 7 days in each distance group (*p* = 0.25). Thirty‐day readmissions from index hospitalization were similar by travel distance categories (11.82%, 9.03%, 10.00%, *p* = 0.76) for those ≤ 40 mi vs. 41–100 mi vs. > 100 mi.

**FIGURE 2:**
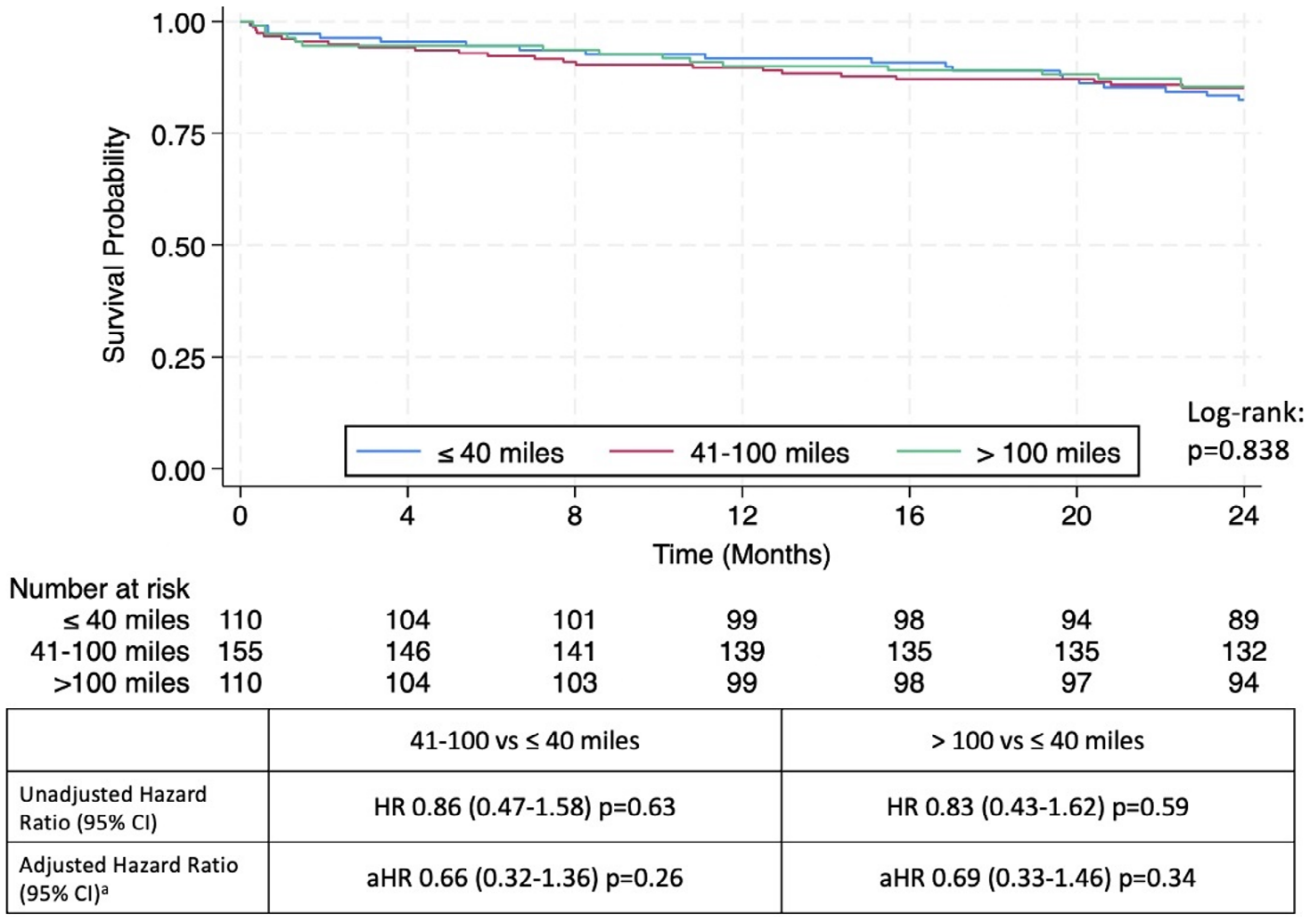
All‐cause mortality by travel distance. Kaplan–Meier survival analysis comparing overall survival by travel distance categories. Survival did not differ significantly between groups (log‐rank *p* = 0.838). Unadjusted and adjusted cox proportional hazards models similarly demonstrated no significant association between travel distance and mortality. CI, confidence interval; HR, hazard ratio; aHR, adjusted hazard ratio. ^a^ Multivariable cox proportional hazards regression adjusted for age, race, year of surgery, cancer stage and type, medical co‐morbidities and indication for colonoscopy.

Among the entire cohort, mortality at 24 months was 17.27%, 14.84%, and 14.55% among Veterans ≤ 40 mi, 41–100 mi and > 100 mi away (*p* = 0.82). There was no statistically significant association between 2‐year overall survival and travel distance categories (Figure 2). When adjusted for covariates and when distance was examined as a continuous variable, the association between travel distance and 2 year mortality was not statistically significant (adjusted HR 0.996 for every 1 mi increase in distance, CI 0.99–1.002).

### 3.5 Exploratory Analysis for Delays in Care

Amongst Veterans who had care delays greater than 60 days for treatment, the most common reasons for delay included need for cardiac clearance or optimization (40.6%), additional testing for oncologic staging procedures (repeat colonoscopy [24.5%] or EUS [26.6%]), or care transition from non‐VA facility (25.0%). Patient specific reasons constituted a minority of the care delays (Table 3).

**TABLE 3:** Factors contributing to initiation of treatment > 60 days after colorectal cancer diagnosis.

Reason for delay	Percent affected^a^
Medical	
Cardiac clearance or optimization (i.e., including need for preoperative PCI/CABG^b^)	26 (40.63%)
Noncardiac physiologic testing or optimization (i.e., PFTs^c^, blood pressure control)	7 (10.9%)
Workup of incidental finding	12 (18.8%)
Other medical problem taking precedence (myocardial infarction, other cancer)	13 (20.3%)
System	
Delay in EUS^d^	17 (26.6%)
Required additional colonoscopy (for confirming diagnosis/location, tattooing, etc.).	15 (24.5%)
Scheduling issues	7 (10.9%)
Care transition from outside facility	16 (25.0%)
Changes in provider decision‐making regarding care plan or delay in tumor board discussion	9 (14.1%)
Patient	
Difficulty contacting patient	7 (10.9%)
Patient desire to delay surgery, unsure about proceeding, etc.	5 (7.8%)
Patient issues keeping appointments	5 (7.8%)

*Note:* All patients who did not initiate treatment within 60 days of diagnosis were reviewed, *N* = 64.

^a^ Percentages do not sum to 100% as many patients had more than one reason for delay.

^b^ PCI = percutaneous coronary intervention, CABG = coronary artery bypass graft surgery.

^c^ PFTs = pulmonary function tests.

^d^ EUS = endoscopic ultrasound.

## 4 Discussion

The findings of this study demonstrate that among Veterans, travel distances > 100 mi—representing the group with the highest proportion of rural residence—are associated with an approximate 11 day difference in time from diagnosis to initiation of oncologic treatment. However, there were no significant differences in treatment timing for Veterans traveling 41–100 mi compared to those within 40 mi. Further, there were no associations between travel distance and other key patient outcomes including length of stay, 30 day readmission or all‐cause mortality. Collectively, these findings suggest that although numerical differences in time to treatment initiation may represent areas for improvement, delays of this magnitude were not associated with measurable differences in the clinical outcomes evaluated in this study within a high‐volume, integrated care system.

Overall time to CRC treatment initiation for Veterans in our study was comparable to that reported in other studies in academic and non‐VHA settings [28, 29, 30, 31]. Importantly, there is no universally accepted benchmark for the interval from CRC diagnosis to treatment initiation. Current American Society of Colon and Rectal Surgeons (ASCRS) guidelines recommend proceeding with curative‐intent treatment “without unnecessary delay” but there are no ASCRS or National Comprehensive Cancer Network (NCCN) guidelines that define a specific treatment interval [32, 33, 34]. Notably, our median time to treatment of 33 days falls within the optimal window identified in a prior dose–response meta‐analysis [35]. Our findings are similar to those reported by Scoggins et al. who evaluated a cohort of Medicaid patients and found that the time to initiation of treatment was 14.57 days longer per 100 driving miles [36]. In the broader US population, increased distance from CRC treatment facility has been linked to more advanced disease and increased post‐operative length of stay [37, 38, 39]. Massarweh et al. noted that longer distance to treatment facility was associated with an increased likelihood for metastatic disease, a population excluded from our analysis [38]. Therefore, our results may be conservative for this group. Among Veterans with CRC, Martin et al. found that time to initiation of post‐operative chemotherapy was 10.6 days longer for Veterans living > 100 mi from the hospital compared to < 50 mi [40]. The present study augments the contextual understanding of Veterans' experience of CRC care by examining the preoperative interval.

This study adds valuable perspective by examining the impact of travel distance on CRC care timeliness within the VHA—a unique healthcare setting and patient population. The VHA represents the largest single‐payer healthcare system in the US. Within the Canadian single‐payer, universal coverage healthcare system, urban patients experienced shorter times from diagnosis to surgery for Stages I–III colon cancer as compared to rural and suburban patients [41]. Mooney et al. noted an average travel distance of 61 mi for VA facility users seeking inpatient medical or surgical care, comparable to the 67.9 mi average among our CRC cohort [42]. In the US in general, 77.6% and 85.5% of rural patients must travel over 30 mi to access the nearest colorectal surgeon or surgical oncologist respectively, underscoring the challenges of accessing specialized care [43]. The proportion of patients traveling more than 60 min for surgical care has increased over the last decade [20]. With increasing centralization of cancer care, these findings contribute to a broader understanding of CRC care in different rural populations nationwide.

Although timeliness of care has been identified as a healthcare quality domain, its precise role in CRC outcomes remains under investigation [44]. Some studies report associations between treatment delays and all‐cause mortality in CRC, while others do not [4, 45, 46, 47, 48]. Although no universally accepted treatment interval exists, one meta‐analysis suggests that overall survival declines after delays exceeding 4 weeks [49]. The 11.8 day difference observed in our study falls well within this window, consistent with the absence of outcome differences and underscoring the capacity of the integrated VA system to deliver timely care even for the most geographically distant Veterans. Importantly, not all delays necessarily represent lower‐quality care. Additional pretreatment evaluation, multidisciplinary treatment planning, staging procedures, or medical optimization may appropriately extend the interval to treatment initiation for selected patients, as reflected in our exploratory analysis. The lack of association between travel distance and key outcomes in our study aligns with findings from other high volume centers. Using the US National Cancer Database (NCDB), Raman et al. found no survival difference between urban and rural patients with Stage I–III colon cancer treated at high‐volume centers [48]. In rectal cancer, Xu et al. demonstrated that travel to a high‐volume center may offset travel burden by improving lymph node yield, receipt of neoadjuvant chemotherapy and 30–90 day mortality [50]. Taken together, these findings suggest that although the observed difference in treatment timing represents an opportunity to improve care coordination, its clinical significance may be limited within a high‐volume, integrated healthcare system. While caution should be taken in equating a relationship between distance and timeliness directly with quality of care, outside of clinical outcomes delays in care may still impact patient quality of life, fear, anxiety, and overall experience [51, 52, 53, 54]. Future research should explore whether numerical differences in timeliness translate into other meaningful patient‐centered outcomes. In the absence of a universally accepted benchmark for time to treatment initiation, continued evaluation of treatment timeliness remains important for informing quality improvement initiatives and optimizing colorectal cancer care.

Transitions between non‐VA and VA care were among the most common contributors to increased treatment times in our cohort. This aligns with literature suggesting system‐level factors and care transitions contribute more to CRC delays than patient characteristics [55]. Targeted interventions addressing care coordination at transition points therefore may offer the greatest opportunity to improve timeliness. Consolidation of care within the integrated VA system, rather than fragmentation across systems, may help preserve the quality advantages of high‐volume, integrated cancer care.

This study is limited by its single‐institution, observational design within one geographic catchment area, and inclusion of only Veterans who ultimately underwent colorectal cancer surgery at a single VAMC. The Veteran population is predominantly male and White, which limits generalizability to other patient populations. Sample size may limit statistical power to detect differences in timing and outcomes across groups. Additionally, data on the timing of initial symptoms were unavailable, preventing analysis of how travel distance might influence delays in diagnosis. However, the absence of stage differences at presentation across distance groups suggests that any diagnostic delays, if present, were unlikely to be clinically significant. Travel distance, while a useful proxy for geographic access, does not fully capture actual travel burden, including travel time, transportation mode/availability, road conditions, caregiver support, or other patient‐specific barriers to accessing care. Although rurality was also evaluated using RUCA classifications, neither measure fully captures individual differences in healthcare access. Accordingly, the observed associations should be interpreted as reflecting geographic distance rather than the full spectrum of travel‐related barriers. The study period spanned nearly two decades, during which colorectal cancer management and VA healthcare policies evolved. We accounted for temporal trends by adjusting for year of surgery modeled nonlinearly in all multivariable analyses; however, residual confounding from unmeasured changes in clinical practice or healthcare delivery over time remains possible.

Additional important considerations for the interpretation of these findings include the passage of the CHOICE Act (2014) and Mission Act (2018) which expanded non‐VA care access among Veterans living more than 40 mi from a VAMC [22, 23, 24]. Between 2014 and 2019 approximately 15% of Veteran procedures occurred in non‐VA facilities, typically among younger, White Veterans with lower comorbidity scores [21]. While the ultimate impact of these changes remains unclear, our study focused on timeliness of care for Veterans receiving surgery at the VA to evaluate travel‐related barriers in a single integrated healthcare system. Consequently, Veterans who underwent surgical treatment in the community were not captured in this cohort, and Veterans traveling the greatest distances in our cohort may represent a selected subset of those receiving VA care. This may introduce selection bias with respect to the broader Veteran population and limit generalizability, particularly for Veterans receiving definitive surgical treatment outside the VA. If Veterans most affected by travel barriers preferentially received community‐based surgery, the observed association may underestimate the impact of travel burden among all Veterans with colorectal cancer. Notably, transitions between non‐VA and VA care were among the most common contributors to increased treatment times here, while delays solely within the VA were less frequent. Given these evolving patterns of care, our findings should be interpreted within the context of the pre‐Mission act VA healthcare system. Nevertheless, they establish an important baseline for understanding travel‐related barriers within an integrated VA healthcare system and provide a framework for evaluating how expanded community care has influenced treatment timeliness and care coordination in the contemporary era. As referral patterns and community care integration evolve, future research should further explore how distance and inter‐system care coordination influences different outcomes.

## 5 Conclusions

Among Veterans with CRC, travel distances > 100 mi were associated with longer times to treatment initiation, though no differences in clinical outcomes were observed within the VA system. As cancer care becomes increasingly regionalized, travel distance and wait times will remain important considerations in evaluating access and quality. The VHA continues to deliver high quality cancer care, with outcomes comparable to or surpassing those of non‐VA settings [11, 12, 13]. This study contributes valuable data to inform patient counseling regarding travel and care expectations, highlighting comparable timeliness within 100 mi and similar outcomes across all distance groups. Delays related to additional pretreatment workup and intersystem care transitions represent key targets for improving care efficiency. Rural Veterans traveling the greatest distances may benefit from enhanced care coordination strategies including patient navigation, coordinated scheduling, multidisciplinary collaborative clinics (Surgery, Oncology, Nutrition, Social Work and Clinical Psychology), and telehealth integration.

## Author Contributions

**Emma M. Bradley:** conceptualization, methodology, software, data curation, investigation, validation, formal analysis, visualization, project administration, resources, writing – original draft, writing – review and editing. **Megan Shroder:** validation, data curation, writing – review and editing. **Gretchen Edwards:** methodology, validation, data curation, writing – review and editing. **Christianne L. Roumie:** conceptualization, methodology, investigation, formal analysis, writing – original draft, writing – review and editing, supervision, project administration. **Colleen Kiernan:** conceptualization, writing – review and editing, supervision. **Shannon McChesney:** conceptualization, writing – review and editing. **Robert S. Dittus:** supervision, writing – review and editing, conceptualization.

## Funding

E.B., M.S., and G.E. have been supported by the Veterans Administration Medical Center (VA Quality Scholars Program).

## Conflicts of Interest

The authors declare no conflicts of interest.

## Supporting information


**Table S1:** Adjusted linear regression for time from diagnosis to initiation of treatment based on varying the distance categories for treatment initiation to 20, 60, 80, and 120 day.

**Table S2:** Descriptive relevant comparisons of the cohort before and after 2014 (when passage of the CHOICE act expanded community care access for patients living > 40 mi from the VA). Includes distance categories and number of patient medical comorbidities.

**Table S3:** Select clinical outcomes among distance categories. Includes length of stay, 30‐day readmissions after index hospitalization, and mortality at 2 years.

## Data Availability

The data that support the findings of this study are derived from the Veterans Health Administration (VHA) and contain protected health information. Due to federal privacy regulations, VA data governance policies, and data use agreements, these data cannot be made publicly available. Qualified investigators may request access through the US Department of Veterans Affairs by submitting a proposal for review and approval through the appropriate VA data access and regulatory processes, subject to institutional approvals and data security requirements.
