# Supplementary material for: Timeliness of Colorectal Cancer Care in Veterans: The Role of Travel Distance
Source: Cancer Rep (Hoboken). 2026 Aug 31;9(9):e70663. doi: 10.1002/cnr2.70663 (PMC13529579; doi:10.1002/cnr2.70663)
Supplement: Supplementary file 1 [file CNR2-9-e70663-s001.docx]

| Distance from VAMC | β Coefficient  (Days Difference) | 95% Confidence Interval | p-value |
| --- | --- | --- | --- |
| Ref: ≤20 miles |  |  |  |
| 20-40 miles | -0.46 | -15.38-14.47 | 0.95 |
| >40 miles | 4.67 | -7.66-16.99 | 0.46 |
| Ref: ≤40 miles |  |  |  |
| 40-60 miles | -5.69 | -18.26-6.88 | 0.76 |
| >60 miles | 8.06 | -1.10-17.22 | 0.08 |
| Ref: ≤40 miles |  |  |  |
| 40-80 miles | -0.05 | -10.62-10.52 | 0.99 |
| >80 miles | 8.14 | -1.43-17.71 | 0.10 |
| Ref: ≤40 miles |  |  |  |
| 40-120 miles | 1.92 | -7.42-11.27 | 0.69 |
| >120 miles | 11.49 | 0.31-22.67 | 0.04 |
| Rurality |  |  |  |
| Rural vs Urban | 8.82 | -2.31-19.95 | 0.12 |

**Supplementary Table 1:** Adjusted linear regression for time from diagnosis to initiation of treatment based on varying the distance categories for treatment initiation to 20, 60, 80, and 120 day.
