# Supplementary material for: Timeliness of Colorectal Cancer Care in Veterans: The Role of Travel Distance
Source: Cancer Rep (Hoboken). 2026 Aug 31;9(9):e70663. doi: 10.1002/cnr2.70663 (PMC13529579; doi:10.1002/cnr2.70663)
Supplement: Supplementary file 2 [file CNR2-9-e70663-s002.docx]

**Supplementary Table 3:** Select clinical outcomes among distance categories. Includes length of stay, thirty-day readmissions after index hospitalization, and mortality at 2 years.

|  | Distance from VAMC | | | |
| --- | --- | --- | --- | --- |
|  | 0-40mi | 40-100mi | >100mi | p-value |
|  | (N=110) | (N=155) | (N=110) |  |
| Length of Stay, median days (IQR) | 7 (6-10) | 7 (5-9) | 7 (5-9) | 0.25 |
| Thirty-Day Readmissions, n (%) | 13 (11.82) | 14 (9.03) | 11 (10.00) | 0.76 |
| Mortality at 2 years, n (%) | 19 (17.27) | 23 (14.84) | 16 (14.55) | 0.82 |
