# Supplementary material for: Timeliness of Colorectal Cancer Care in Veterans: The Role of Travel Distance
Source: Cancer Rep (Hoboken). 2026 Aug 31;9(9):e70663. doi: 10.1002/cnr2.70663 (PMC13529579; doi:10.1002/cnr2.70663)
Supplement: Supplementary file 3 [file CNR2-9-e70663-s003.docx]

**Supplementary Table 2:** Descriptive relevant comparisons of the cohort before and after 2014 (when passage of the CHOICE act expanded community care access for patients living >40 miles from the VA). Includes distance categories and number of patient medical comorbidities.

|  | Year of Treatment | | |
| --- | --- | --- | --- |
|  | Prior to 2014 | After 2014 | p-value |
|  | (N=290) | (N=85) |  |
| Travel Distance, n (%) |  |  | 0.34 |
| 0-40 miles | 81 (27.93%) | 29 (34.12%) |  |
| 40-100 miles | 119 (41.03%) | 36 (42.35%) |  |
| >100 miles | 90 (31.03%) | 20 (23.53%) |  |
| Average Number of Medical Comorbidities, mean ± standard deviation | 1.49 ± 1.19 | 1.56 ± 1.28 | 0.75 |

^a^ Includes chronic kidney disease, chronic obstructive pulmonary disease, congestive heart failure, cardiovascular disease, diabetes and obesity (as defined in the Methods section).
